# Peptidylarginine deiminase type 2 is over expressed in the glaucomatous optic nerve

**Published:** 2010-08-17

**Authors:** Thamara A. Cafaro, Stefanía Santo, Lucena A. Robles, Nicolás Crim, Julio A. Urrets-Zavalia, Horacio M. Serra

**Affiliations:** 1 CIBICI, Faculty of Chemistry, National University of Córdoba, Argentina; 2 University Clinic Reina Fabiola, Universidad Católica de Córdoba, Córdoba, Argentina

## Abstract

**Purpose:**

To determine levels of Peptidyl arginine deiminase 2 (PAD2) and its product protein-bound citrulline in cadaver eyes that suffered from normal tension glaucoma (NTG) compared to primary open angle glaucoma (POAG), and controls.

**Methods:**

Western analysis, ELISA, and immunohistochemical analysis were performed with human tissues.

**Results:**

We report over expression of PAD2 and higher levels of its product protein-bound citrulline in the optic nerve of normal tension glaucoma patients (NTG).

**Conclusions:**

This is the first report demonstrating that like in POAG, NTG also possesses elevated levels of both PAD2 and protein-bound citrulline.

## Introduction

Glaucomas are a group of irreversible blinding diseases that are divided into two categories, primary when ascribed to no previous injury and illness and secondary when it can be attributed to a previous incident [1]. Normal tension glaucoma (NTG) in either category is a type of glaucoma where intraocular pressure (IOP) of the subjects remains within the normal levels [2]. Primary open angle glaucoma (POAG) is usually the most prevalent form. The intraocular pressure is normally increased in glaucoma due to an imbalance between aqueous humor production and outflow and is thought to be a major risk factor for development of glaucoma [1,3]. Aqueous humor is a clear fluid that serves vital functions like nourishing the cornea and the tissues in the anterior chamber [4]. Glaucoma is characterized by damage to the optic nerve termed glaucomatous optic neuropathy (GON). The optic nerve damage is clinically measured as changes in visual acuity using static perimetry. Damage to the optic nerve is usually late onset and progressive in nature.

The pathologic process of glaucoma remains poorly understood. Understanding molecular changes in glaucoma will allow gaining an insight into the pathologic process and may provide value in predicting glaucoma susceptibility. The optic nerve damages are difficult to assess as those changes locally occur in the cells in the optic nerve. There is no good spontaneous animal model of glaucoma available that enables extensive invasive analyses in vivo and in vitro to simultaneously determine clinical disease progression while enabling molecular analysis although genomic and proteomic analyses on human cadaver tissues have provided some insight. Whereas the changes in mRNA levels unrevealed by genomics does not always correlate with functional proteins they provide important insight. Proteomic analysis of primary open angle glaucoma (POAG) cadaver tissues and subsequent analysis has revealed elevated levels of peptidylarginine deiminase type 2 (PAD2) and its product protein-bound citrulline [5]. However, levels of PAD2 and that of protein-bound citrulline have not been investigated in cadaver eyes that had normal tension glaucoma. We present our findings of elevated levels of PAD2 and protein-bound citrulline in normal tension glaucoma patients. Our results also further corroborate previous proteomic findings in POAG patients.

## Methods

### Tissue procurement

Donor eyes from normal (control), POAG, and NTG cadavers were enucleated within 72 h of death and obtained from the National Disease Research Interchange, Philadelphia, PA and the Florida Lions Eye Bank, Largo Medical Center Hospital, Largo, FL. The available medical and ophthalmic histories of cadaver eyes were recorded (Table 1). The controls eyes lacked optic neuropathy. Glaucomatous and age-matched normal eyes from donors were used in this study (Table 1). Research was conducted following the tenets of the Declaration of Helsinki. The optic nerve was dissected from the globe. A small incision was made with a scalpel and the entire retinal tissue attached to the optic nerve was excised with a pair of scissors. The dissected optic nerve tissue comprising optic nerve head and the stock (usually 5–7 mm) was subjected to removal of fat by trimming the outer part of the stock and subjected to subsequent protein extraction. The globe was subjected to other analysis not related to research mentioned here.

**Table 1:** Details of tissue donors.

**Age**	**Gender**	**Race**	**Diseases**	**C/D ratio**	**Mean defect**	**IOP**
**Control donors**						
62	M	Caucasian	Cardiac arrest	N/A	N/A	11
70	F	Caucasian	Cardiac arrest	N/A	N/A	N/A
66	M	Caucasian	Cardiac arrest	N/A	N/A	N/A
77	F	Caucasian	Cardiac arrest	N/A	N/A	14
75	M	Caucasian	Cardiac arrest	N/A	N/A	N/A
74	F	Caucasian	Cardiac arrest	N/A	N/A	N/A
**Glaucoma donors**						
65	F	Caucasian	Cardiac arrest	1	−11	14.5
74	M	Caucasian	Cardiac arrest	0.7	−12	16
72	F	Caucasian	Cardiac arrest	0.8	−10	14
80	M	Caucasian	Cardiac arrest	0.6	−11	16.5
74	F	Caucasian	Cardiac arrest	0.8	−10	16
78	F	Caucasian	Cardiac arrest	0.7	−9	14
72	F	Caucasian	Cardiac arrest	0.8	−7	22
75	M	Caucasian	Cardiac arrest	0.7	−8	24

In the table, C/D ratio=Cup to disk ratio; GHT=Glaucomatous hemifield test for all glaucoma donors were outside normal limit; All donor eyes used in this study that had records of automated static perimetry used a full threshold 24–2 test procedure. All controls had a visual acuity of 20/30 or better, no cataract, glaucoma or any other known eye disease. Mean defect have been rounded off to single digit numbers. The IOP measurement refers to last known measurement before death.

### Western analyses

Protein was extracted from the optic nerve by homogenization in 125 mM Tris-Cl buffer, pH 7.0, containing 100 mM NaCl, 5 mM dithiothreitol, 0.1% Triton X-100, 0.5% Tween-20, and 0.5% SDS. Insoluble material was removed by centrifugation (8,000× g for 5 min), and soluble protein quantified by the Bradford assay. Western analyses were performed with 5 µg of protein, 4%–20% gradient gels (Invitrogen Inc., Carlsbad, CA), electroblotted to PVDF membrane and probed with antibodies: rabbit polyclonal antibody against PAD2 (Abcam Inc., Cambridge, MA), mouse antibody to glyceraldehyde-3-phosphate dehydrogenase (GAPDH; Millipore Corporation, Billerica, MA) and against protein-bound monoxime modified citrulline (Millipore Corporation). For quantitative western analyses, secondary antibodies conjugated with horseradish peroxidase were used.

### Enzyme linked immunosorbent assay (ELISA) analyses

About 5 μg of purified in vitro deiminated BSA (BSA) or 5 μg of protein extract was centrifuged at 8,000× g for 5 min and 100 μl clear solution was transferred to wells in a multiwell plate (Costar plates-e Bioscience Inc., San Diego, CA) and incubated for 20 min at room temperature. Recombinant cellular retinaldehyde binding protein (CRALBP) produced in *E. coli* was used as non-deiminated control. The recombinant CRALBP and in vitro fully deiminated BSA (5 μg) was used as non-deiminated and fully deiminated control, respectively. Proteins were incubated with 40 μl/well of 1:1 reagent A and B from a citrulline kit (17–347; Millipore Corporation) at 37 °C for 90 min. The supernatant was discarded and the plate was washed with PBS. The plates were blocked with 1% BSA for 1 h, washed with PBS, and incubated for 1 h with rabbit polyclonal antibody against modified citrulline. After washes with PBS, plates were incubated with the secondary antibody coupled with alkaline phosphatase for 1 h, washed with PBS and incubated with phosphatase substrate (100 μl/well) in diethanolamine buffer pH7.5. The absorbance was then measured at 405 nm on a plate reader. For detection of PAD2, the reagent A and B incubation step was omitted and a rabbit polyclonal antibody against PAD2 (ab16478; Abcam Inc.) was used for detection using an identical procedure. Purified CRALBP alone and recombinant purified PAD2 (5 μg) on purified CRALBP under identical conditions served as negative and positive control respectively. ELISA analyses results are expressed as mean±standard deviation. Statistical analysis was performed for each sample at every experimental point shown, compared to 0.0 using the two tailed one-sample *t*-test and was found significantly different from 0.0 at each time point by the one-sample *t*-test: p<0.05.

### PAD2 activity assay

Activity of PAD2 was measured using a monooxime treatment followed by assessment of optical density using suitable modification or previous procedures [6]. Briefly, the reaction mixtures containing 100 mM Tris-HCl, pH 7.5, 10 mM CaCl_2_, 2.5 mM DTT, 10 benzoylarginine ethyl ester (BAEE), and an appropriate amount of protein extract (5 μg/sample) in a final volume of 50 μl were incubated at 37 °C for 3 h. The reaction of enzyme activity was stopped by adding 10 μl of 5 M perchloric acid and the perchloric acid-soluble fraction was subjected to 150 μl of carbidino detection reagent, which was assembled freshly from its two components with one of part A containing 0.5% diacetyl monoximine and 0.01% thiosemicarbazide added to two of parts B containing 0.25 mg of FeCl_3_/ml in 24.5% sulfuric acid (H_2_SO_4_) and 17% phosphoric acid (H_3_PO_4_). The reaction was mixed vigorously and heated at 65 °C for 5 min. After heating, samples were cooled to room temperature and the absorbance was measured at 435 nm in a spectrophotometer. As a control, purified recombinant cellular retinaldehyde binding protein (CRALBP) prepared in bacteria was subjected to recombinant PAD2 treatment and re-purified.

### Immunohistochemistry

Histological evaluations were made following previously published protocols for protein-bound citrulline. The 4% paraformaldehyde in phosphate buffer fixed and subsequently paraffin embedded anterior eye sections (about 10 µm) were prepared and stained with antibodies to PAD2 and protein-bound citrulline. Presence of protein-bound citrulline was verified using fluorescence analyses using Alexa 488 and Alexa 594 coupled secondary antibodies (Invitrogen Molecular Probes, Eugene, OR). To ensure identical processing and uniform exposure, controls (without antibody and primary antibody treated sections) were examined side by side on the same slide. Fluorescence images were obtained using a Leica TCS-SP5, laser scanning confocal microscope (Leica, Exton, PA). A series of 1 µm xy (en face) images were collected and summed for an image representing a three-dimensional projection of the entire 10 µm section. Confocal microscopic panels were composed using Adobe Photoshop 8.0.

## Results

**Figure 1:**
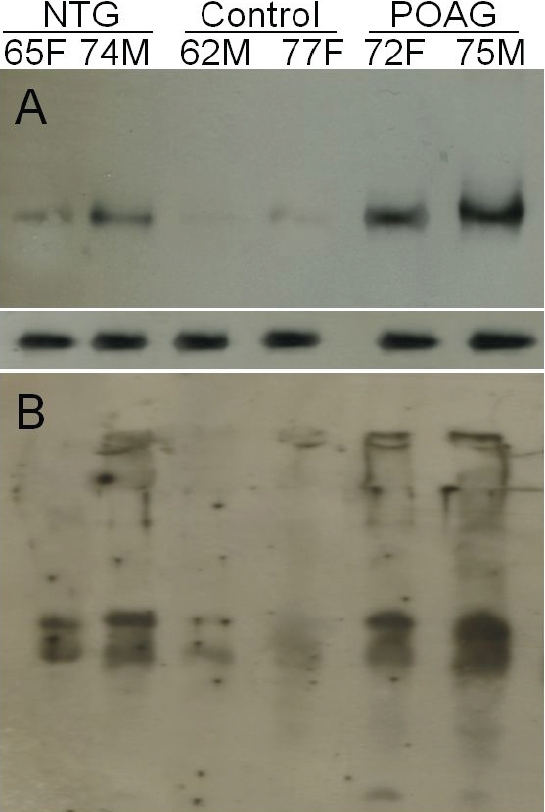
Elevated PAD2 and protein-bound citrulline immunoreactivity in glaucomatous optic nerve. **A**: Representative western analyses with monoclonal anti-PAD2 of protein extracts from human optic nerve tissue (~25 µg per lane) demonstrating the presence of elevated levels of PAD2 was detected in glaucomatous tissues. Lower panel shows the GAPDH immunoreactivity indicating equal protein loading. **B**: Western analyses with rabbit polyclonal antibody to citrulline (25 µg protein per lane). Prior to applying antibody, membrane with transferred proteins was treated with 2,3-butanedione monooxime and antipyrine in a strong acid environment to modify the protein-bound citrullines to enable the detection with the antibody. Proteins were extracted from the optic nerve of Caucasian cadaver donor eyes: age and gender are indicated.

**Figure 2:**
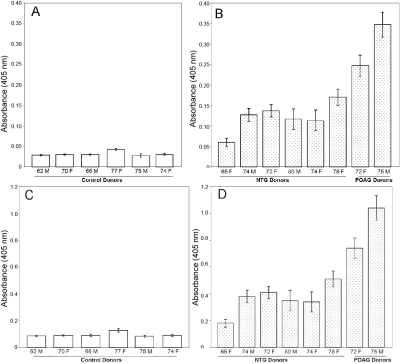
The PAD2 and level of deimination using ELISA analyses as described in methods. **A** and **B**: PAD2 level represented by hollow bars and dotted bars for control and glaucomatous donors. **C** and **D**: Level of deimination are represented by hollow bars and dotted bars for control and glaucomatous donors respectively. The results are standard deviation of three independent experiments.

**Figure 3:**
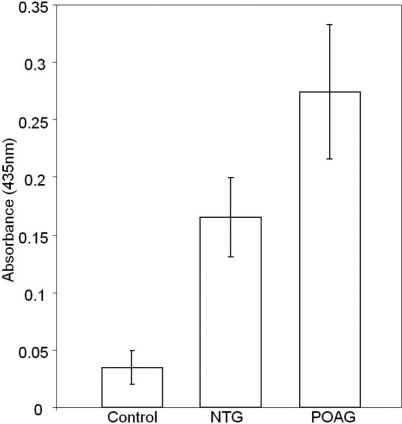
Elevated PAD2 activity in glaucomatous optic nerve. PAD activity was measured as described in the methods and absorbance at 435 nm has been shown.

**Figure 4:**
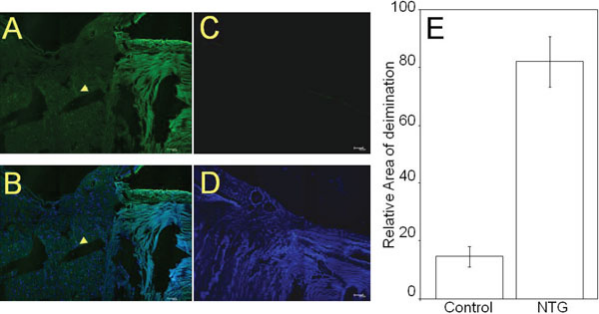
Representative immunohistochemical analyses of NTG (70 F) and control (72F) donor optic nerve sections. **A**: Immunoreactivity for protein-bound citrulline detected by a rabbit polyclonal antibody to citrulline after monoxime modification as described in methods. **B**: Merged image of anti-citrulline with DAPI. **C**: Control donor section probed for immunoreacitivty with anti-citrulline. **D**: Merged image of anti-citrulline and DAPI for control donor section. **E**: Densitometric analysis of immunohistochemical detection of protein-bound citrulline in NTG eyes. The data was analyzed using Image J software.

Elevated PAD2 was detected in normal tension glaucoma donors as well as in POAG donors (Figure 1A and Figure 2A,B). Commensurate with elevated PAD2, elevated protein-bound citrulline (Figure 1B and Figure 2C,D) was also detected in NTG optic nerve compared to control. Activity measurement showed elevated PAD2 in protein extract derived from NTG and POAG donors compared to control (Figure 3). Elevated PAD2 activity is commensurate with elevated PAD2 immunoreactivity as well as that for protein-bound citrulline (Figure 1). We also investigated protein-bound citrulline immunoreactivity (product of PAD2 activity) using immunohistochemistry in optic nerve sections of NTG eyes (Figure 4A,B). The immunohistochemical comparison of protein-bound citrulline immunoreactivity, commensurate with western (Figure 1B) and ELISA (Figure 2) analyses showed elevated immunoreactivity in the NTG compared to control eyes. All sections subjected to immunohistochemical analyses were also subjected to relative intensity measurement with after normalization of measurement area which showed relatively elevated levels of protein-bound citrulline in NTG eyes compared to controls (Figure 4C).

## Discussion

We report here for the first time, elevated PAD2 immunoreactivity (Figure 1A), activity (Figure 3) and immunoreactivity for protein-bound citrulline (Figure 4) in normal tension glaucoma eyes compared to controls. While elevated PAD2 and protein-bound citrulline has been shown in POAG [5], the same has not been previously investigated in NTG. PAD2 catalyzes conversion of protein-bound arginine into citrulline (but from free arginines) termed deimination [7]. There exists no known enzyme to reverse the posttranslational deimination rendering this modification a long-term modification that is reversed only with degradation of proteins [8]. Previously, PAD2 and elevated deimination has been implicated in neurodegenerative changes [9,10] and in kainic-acid evoked neurodegeneration [11]. Although unbiased proteomic analyses showed PAD2 and elevated levels of PAD2 and deimination, its role in glaucomatous neurodegeneration remains unclear. It appears that in response to environmental stimuli, different cells of neuronal systems express different levels of PAD2, whereas the PAD2 expression undergo a decrease in response to certain environmental stimuli in neurons that of other cell types, for example, in astrocytes it increases [8]. Further investigation will reveal the detailed mechanistic insight of the role of relatively long-term posttranslational modification of deimination in the glaucomatous neurodegeneration. Our discovery of elevated deimination in NTG suggests possible underlying common mechanistic features in all glaucomatous optic neuropathies.
